# EHMTI-0160. New questionnaire – impact of migraine, tension-type headache and neck pain: a validation study

**DOI:** 10.1186/1129-2377-15-S1-D58

**Published:** 2014-09-18

**Authors:** LS Krøll, C Sjödahl Hammarlund, G Gard, RH Jensen

**Affiliations:** 1 Department of Neurology, Danish Headache Center, Copenhagen, Denmark; 2 Department of Health Sciences, Lund University, Lund, Sweden

## Introduction

Migraine often include co-existing tension-type headache (TTH) and neck pain (NP). Currently no questionnaires cover these co-morbidities. A new questionnaire “Impact of Migraine, Tension-Type Headache and Neck Pain” (Impact M-TTH-NP) was therefore needed.

## Aim

To determine face and content validity of the newly developed Impact M-TTH-NP.

## Methods

Nine migraine patients with co-existing TTH and NP participated in group interviews. Content validity was assessed by 13 headache experts. The Content Validity Index (CVI) at item (I-CVI) and at scale level (S-CVI/Ave) was used together with the Average Deviation (AD) index to assess interrater agreement.

## Results

Impact M-TTH-NP showed acceptable face validity. Of 78 items twelve were revised and one was added based on group interviews and experts review. Seventy two items (92%) obtained I-CVI ≥ 0.78 (range 0.78-1.00) indicating excellent content validity, 71 items (91%) obtained acceptable AD index. Nine items did not meet either the limit for excellent I-CVI and/or acceptable AD index. The overall S-CVI/Ave was 0.92 indicating an excellent content validity.

## Conclusions

The Impact M-TTH-NP questionnaire showed acceptable face validity and excellent content validity and can be recommended for clinical studies of patients with migraine and co-existing TTH and NP.

No conflict of interest.

